# Effect of AQP9 Expression in Androgen-Independent Prostate Cancer Cell PC3

**DOI:** 10.3390/ijms17050738

**Published:** 2016-05-14

**Authors:** Qiwei Chen, Liang Zhu, Bo Zheng, Jinliang Wang, Xishuang Song, Wei Zheng, Lina Wang, Deyong Yang, Jianbo Wang

**Affiliations:** 1 Department of Urology, First Affiliated Hospital of Dalian Medical University, Dalian 116021, China; drcqw21@gmail.com (Q.C.); zhengboll@126.com (B.Z.); wangjinliang1114@163.com (J.W.); songxishuang@dlmedu.edu.cn (X.S.); zhengwei1025y@163.com (W.Z.); leon661112@163.com (L.W.); 2 College of Basic Medical Science, Dalian Medical University, Dalian 116044, China; zhuliang0210@sina.com

**Keywords:** prostate cancer, aquaporin 9, RNA interference, proliferation, apoptosis, invasion, ERK signaling pathway

## Abstract

It is known that aquaporin 9 (AQP9) in the prostate was strictly upregulated by androgen and may represent a novel therapeutic target for several cancers, but whether AQP9 plays a role in the regulation of androgen-independent prostate cancer still remains unclear. In the present study, AQP9 was determined in prostate cancer and adjacent cancer tissues; AQP9-siRNA was applied to silencing AQP9 in androgen-independent prostate cancer cell PC3 cell line. Western blot and flow cytometry analysis were employed to detect changes in related-function of control and AQP9-siRNA groups. The results showed that AQP9 is significantly induced in cancer tissues than that in adjacent cancer tissues. Moreover, knockdown of AQP9 in PC3 androgen-independent prostate cancer cell prostate cancer cells increased inhibition rates of proliferation. In addition, knockdown of AQP9 resulted in a significant decrease in the expression of the Bcl-2 and with a notable increase in the expression of Bax and cleaved caspase 3, indicated that AQP9 knockdown promoted apoptosis in prostate cancer cells. From wound healing assay and matrigel invasion, we suggested that AQP9 expression affects the motility and invasiveness of prostate cancer cells. Moreover, In order to explore the pathway may be involved in AQP9-mediated motility and invasion of prostate cancer cells, the phosphorylation of ERK1/2 was significant suppressed in AQP9 siRNA-transfected cells compared with that in control cells, suggesting that AQP9 is involved in the activation of the ERK pathway in androgen-independent prostate cancer cells.

## 1. Introduction

Prostate cancer (PCa) is a leading cause of cancer-related death in Western countries [1]. Localized prostate tumors can be treated and are not a main cause for patient death. However, progression to androgen-independent or metastatic states is remains a clinical challenge [2].

The aquaporin (AQP) family is a membrane protein involved in the selective transport of water across cell membranes. Several subsets of AQPs also transport small molecules such as glycerol and urea [3]. AQPs are critical in mammalian physiology and sometimes involve in other functions, for instance, neural signal transduction and fat metabolism [4,5].

Only recently, the role of AQP in tumor pathogenesis has been identified [6,7]. Tumor cells types express AQPs and a positive correlation exists between histological tumor grade and the AQP expression as compared to normal tissues [8,9]. We previously revealed that AQP9 expression significantly decreased after castration and recovered to the level of the control by androgen replacement from two weeks after castration *in vivo* and *in vitro* [10], therefore, we indicated that AQP9 expression in the prostate was strictly upregulated by androgen. However, when cancer cells developed to androgen-independent, the role of AQP9 in cancer cells remains unclear. In this report, we addressed the functional role of AQP9 in androgen-independent prostate cancer cells invasion, tumor growth and metastasis, and providing an underlying mechanisms of the role of AQP9 in PCa progression.

## 2. Result

### 2.1. Aquaporin 9 (AQP9) Expression in Prostate Cancer Cells

**Figure 1:**
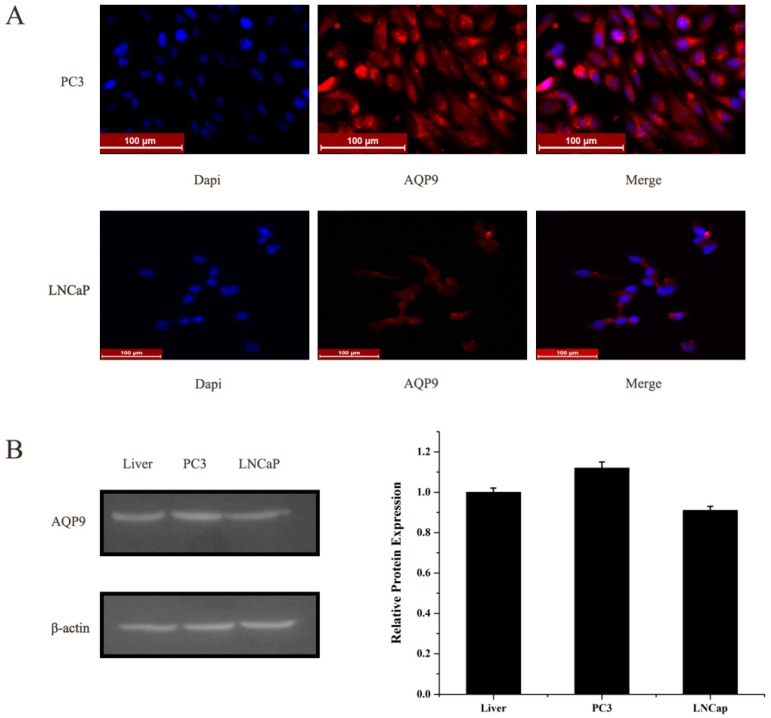
(**A**) Expression of AQP9 in PC3 and LNCap cells was analyzed by immunofluorescence; (**B**) expression of AQP9 in PC3 and LNCap cells was determined by Western blot. The mean of AQP9/β-actin expression in liver set as 1.0. Data were based on three independent experiments, and shown as mean ± SD (standard deviation).

**Figure 2:**
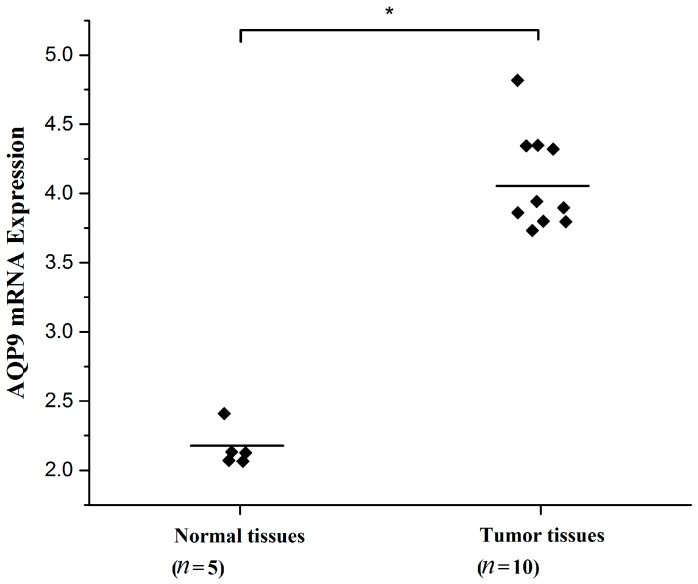
AQP9 expression was significantly increased in prostate cancer tissues when compared with the adjacent tissues of patients from GEO dataset GSE55945 (*p* < 0.05 as compared with control). Bars represent means, * *p* < 0.05.

We first evaluated the expression level of AQP9 in two prostate cancer cells PC-3, LNCap by immunofluorescence, and Western blot. AQP9 immunoreactivity was identified mainly in the cytoplasm of cells (Figure 1A). Moreover, in Western blot analysis we treat normal liver tissue as positive control which was reported before [11], and results revealed that two cell lines, PC-3 and LNCap, showed AQP9 protein expression (Figure 1B). We then analyzed data of prostate cancer patients from GEO (Gene Expression Omnibus) dataset (Access ID: GSE55945) and found that AQP9 expression significantly increased in prostate cancer tissues compared with the adjacent tissues of patients (Figure 2).

### 2.2. Knockdown of AQP9 Suppressed the Proliferation of Prostate Cancer Cells

**Figure 3:**
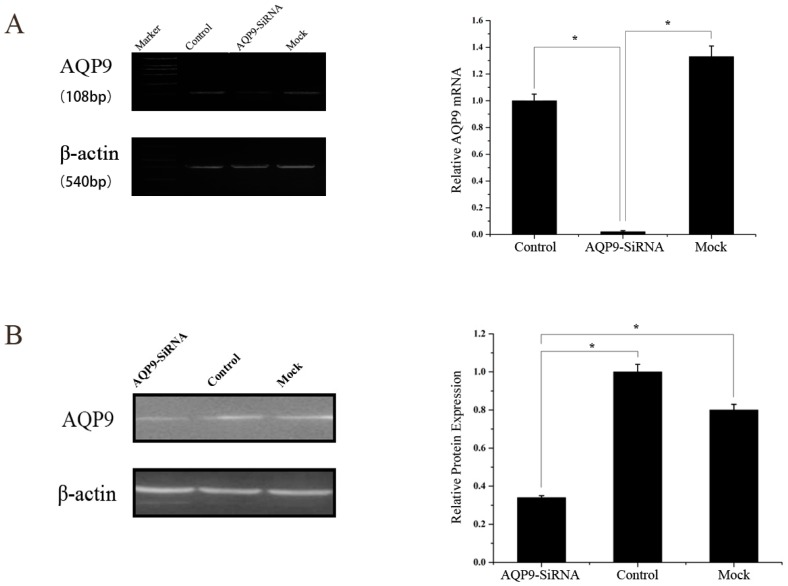
Expression of AQP9 in PC3 cells, AQP9-siRNA and Mock were analyzed by real-time-PCR (**A**) and Western blot (**B**) (*p* < 0.05 as compared with control). △CT AQP9/β-actin (control PC3) was 6.348. Control: wild-type cells; AQP9-siRNA: cells transfected with AQP9 specific small interfering RNA; Mock: cells only treated with Lipofectamine 2000. The mean of AQP9/β-actin expression in PC-3 cells set as 1.0. Data were based on three independent experiments, and shown as mean ± SD. * *p* < 0.05 as compared with control.

**Figure 4:**
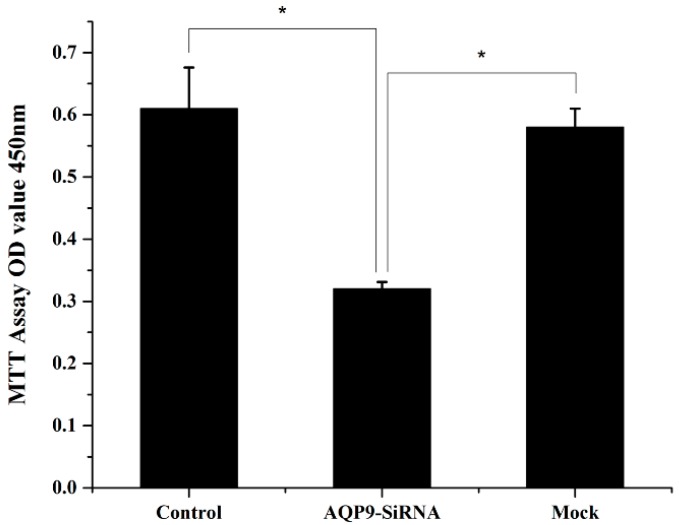
Cell proliferation was detected 24 h after specific small interfering RNA in cells (*p* < 0.05 as compared with control). Control: wild-type cells; AQP9-siRNA: cells transfected with AQP9 specific small interfering RNA; Mock: cells only treated with Lipofectamine 2000. Data were based on three independent experiments, and shown as mean ± SD, * *p* < 0.05.

To investigate the functions of AQP9 on prostate cancer, we knockdown its expression by RNA interference (RNAi) [12]. PC-3 cell line was androgen-independent prostate cancer cell, therefore, we selected PC-3 initially transfected with AQP9 siRNA, and the knockdown efficiency was observed using RT-qPCR and western blot analysis (Figure 3A,B). Knockdown of AQP9 resulted in decreased cell growth rate compared with corresponding control (Figure 4). Thus, we suggested that AQP9 had proliferation-promoting properties in prostate cancer cells. 

### 2.3. Knockdown of AQP9 Induced Apoptosis in Prostate Cancer Cells

**Figure 5:**
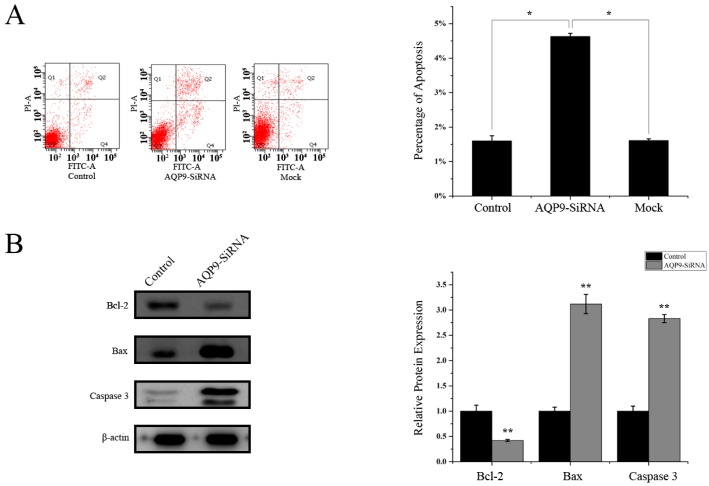
(**A**) Cells were double-stained with Annexin V-FITC/PI and apoptosis rates was analyzed using flow cytometry (*p* < 0.05 as compared with control); (**B**) protein levels of cleaved caspase 3, Bax, and Bcl2 were detected by Western blot. Data were shown as mean ± SD (*p* < 0.05 as compared with control). The mean of targeted protein/β-actin expression in PC-3 cells set as 1.0.Control: wild-type cells; AQP9-siRNA: cells transfected with AQP9 specific small interfering RNA; Mock: cells only treated with Lipofectamine 2000. * *p* < 0.05; ** *p* < 0.01.

We then determined the possible effect of AQP9 knockdown on apoptotic function. As shown in Figure 5A. Flow cytometry analysis revealed that knockdown of AQP9 in PC-3 cells significantly induced cell apoptosis compared with control and mock (4.63% ± 0.09% *vs.* 1.60% ± 0.15% *vs.* 1.61% ± 0.05%). Western blot was then performed to detect apoptosis-related proteins. As shown in Figure 5B, knockdown of AQP9 resulted in a significant reduction in the level of the anti-apoptotic protein Bcl-2 with a notable increase in the level of pro-apoptotic protein Bax and cleaved caspase 3. These results indicated that AQP9 knockdown promoted apoptosis in prostate cancer cells.

### 2.4. Silencing of AQP9 Inhibited the Migration and Invasion of Prostate Cancer Cells

**Figure 6:**
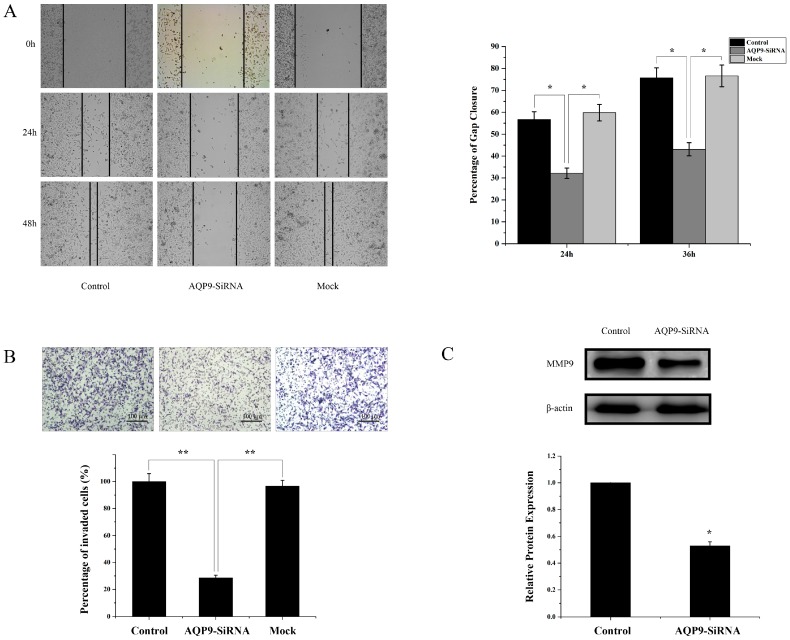
(**A**) Confluent cell monolayers were wounded with a pipette tip. Representative images were captured at 0 h, 24 h, and 36 h. The wound closure was quantified and normalized to that of control cells (*p* < 0.05); Cell invasion was analyzed in Matrigel-coated transwell chambers. Representative images were shown in (**B**); invaded cells were counted in four selected fields (total space is 2 mm^2^) (*p* < 0.05 as compared with control); the total number of invaded cells from the control group set as 100%; (**C**) MMP9 protein expression was examined by Western blot. The mean of MMP9 protein/β-actin expression in PC-3 cells set as 1.0. Control: wild-type cells; AQP9-siRNA: cells transfected with AQP9 specific small interfering RNA; Mock: cells only treated with Lipofectamine 2000. Data were based on three independent experiments, and shown as mean ± SD. * *p* < 0.05; ** *p* < 0.01, Bars, 100 µm.

A wound healing assay and matrigel invasion were then conducted with control cells, AQP9 siRNA-transfected cells, and mock cells. The results showed that silencing of AQP9 inhibited the motility of PC-3 cells (Figure 6A). The effect of AQP9 on invasion of prostate cancer cell was further investigated. The results demonstrated that AQP9 siRNA-transfected cells had lower invasion capabilities compared with the control cells (Figure 6B). MMP (matrix metalloprotein) family play an important role in invasion and metastasis of cancer cells; therefore, the expression of MMP9 between control and AQP9-siRNA were conducted. Western blot analysis showed that silencing of AQP9 downregulated the expression of MMP9 (Figure 6C). These results suggested that AQP9 expression affects the motility and invasiveness of prostate cancer cells.

### 2.5. ERK Pathway Is Required for AQP9-Mediated Motility and Invasion

**Figure 7:**
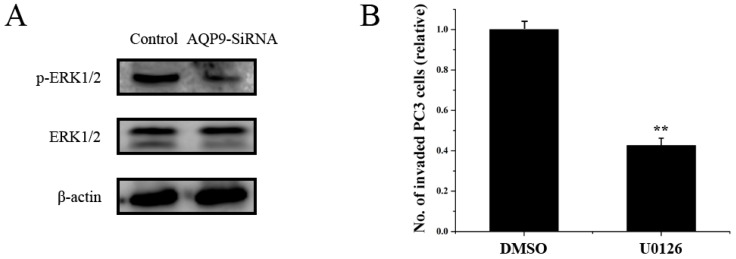
Cells were transfected with siAQP9. (**A**) After 48 h, Western blotting was performed to detect the phosphorylation of ERK1/2 (*p* < 0.05 as compared with control). The phosphorylation of ERK1/2 was markedly suppressed in AQP9 siRNA-transfected cells compared with that in control cells. Control: wild-type cells; AQP9-siRNA: cells transfected with AQP9 specific small interfering RNA; (**B**) after PC3 cells were treated with U0126 (20 µM) or DMSO vehicle, an invasion assay were performed to compare the invasion abilities. The mean number of invaded cells in control group set as 1.0. Data were based on three independent experiments, and shown as mean ± SD, ** *p* < 0.01.

In order to explore which pathways may be involved in AQP9-mediated motility and invasion of prostate cancer cells, the activation of ERK1/2 in PC-3 cells was examined. [13,14]. As shown in Figure 7A, the phosphorylation of ERK1/2 was significant suppressed in AQP9 siRNA-transfected cells compared with that in control cells, Moreover, U0126 was used to suppress the activation of the ERK, in accordance with the result, U0126 treatment inhibited the invasion of PC-3 cells (Figure 7B), suggesting that AQP9 is involved in the activation of the ERK pathway in prostate cancer cells. 

## 3. Discussion

AQP9 is a typical aquaglyceroporin transporting water, glycerol, and urea that plays a major role in fluid homeostasis in normal tissues [15], Recently, some studies reported that AQP9 expression might be involved in the development of several cancers, including epithelial ovarian tumors [16] and glioblastoma [17]. In addition, recent reports have shown that AQP9, a broadly-selective neutral solute and water channel, is abundant in the male reproductive tract and that the expression is regulated by androgens [18,19]. Moreover, our previous work have revealed that AQP9 expression in the prostate was strictly upregulated by androgen both *in vivo* and *in vitro* [10]. However, little is currently known regarding the function of AQP9 in androgen-independent prostate cancer cells. In this work, AQP9 was shown to be expressed in human prostate cancer cells, and AQP9 was silenced in androgen-independent prostate cancer cell line PC-3. After treatment, tumor proliferation was inhibited, as indicated by MTT (3-(4,5-dimethylthiazol-2-yl)-2,5-diphenyltetrazolium bromide) assay. Moreover, we provided evidence that silencing of AQP9 in PC3 cells significantly induced cell apoptosis compared with control cells and mock. Meanwhile, AQP9 knockdown downregulated bcl-2 expression and induced activation of bax and caspase-3, suggesting that AQP9 knockdown reduced proliferation and increased apoptosis which contributed to its suppressive effect on tumor growth.

The present study demonstrated that AQP9 knockdown in PC3 cells significantly decreased their invasion and motility capability; however, the exact pathway that AQP9 may regulate in prostate cancer remains unclear. Therefore, we analyzed the possible pathway with Western blot, the ERK signaling pathway has been considered as a promoter of tumor progression, EMT (Epithelial to Mesenchymal Transition) and invasion [7,20,21,22], AQP9 was shown to promote the activation of ERK1/2 in PC-3 prostate cancer cells, indicating that a possible role for the ERK pathway in AQP9-mediated motility and invasion. Moreover, we addressed the important role of AQP9 in prostate cancer progression to androgen-independent. 

MMP family enhance tumor invasion and metastasis via degradation of the extracellular matrix [23]. Moreover, MMP9 appears to play a key role in promotion of invasion and metastasis in prostate cancer [24,25]. Our present study demonstrated that AQP9 regulates the expression of MMP9 in prostate cancer cells. Moreover, the results showed that inhibition of the ERK pathway decreased the expression of MMP9 in prostate cancer cells, suggesting that the ERK pathway is involved in AQP9-mediated MMP9 expression in prostate cancer cells.

It should be noted that few kinds of androgen-independent prostate cancer cells was the limitation of our study. Some clinical trials are required. In addition, AQP9 is expressed in various organs with important physiological functions. Aquaporin 9 inhibition therapy might lead to some potential damage to other organs and induce serious adverse reactions, which should be considered in future investigations.

## 4. Materials and Methods

### 4.1. Cell Culture and Reagents

PC3, LNCap cells were purchased from American Type Culture Collection (Rockville, MD, USA). PC3 cells were grown in F12 Medium (Life Technology, Carlsbad, CA, USA) with 10% fetal bovine serum (FBS, Life Technology) and 1% antibiotic (penicillin/streptomycin, Life Technology). LNCap cells were grown in RPMI-1640 (Life Technology) with 10% fetal bovine serum (FBS, Life Technology) and 1% antibiotic (penicillin/streptomycin, Life Technology). All cell lines were cultured in an incubator with 5% CO_2_ at 37 °C. Antibodies were AQP9 (Santa Cruz Biotech sc-14988, CA, USA), Bcl-2 (Cell Signaling, Danvers, MA, USA), Bax (Cell Signaling), Cleaved Caspase-3 (Cell Signaling), GAPDH (Santa Cruz Biotech, Santa Cruz, CA, USA), extracellular signal-regulated kinase 1/2 (ERK1/2) (Cell Signaling), phospho-ERK1/2 (Cell Signaling), MMP9 (Proteintech, Chicago, IL, USA). U0126, a specific inhibitor of MEK1/2 was obtained from Beyotime (Shanghai, China). For immunofluorescence labelling, the secondary antibody was rhodamine from Invitrogen (Carlsbad, CA, USA).

### 4.2. Quantitative Real-Time PCR and Western Blot

Cells were harvested and total RNA was isolated using TRIzol^®^ reagent (Invitrogen Life Technologies). qPCRs were carried out as follows: 95 °C for 5 min; 95 °C for 15 s; and 60 °C for 1 min, for 40 cycles. For quantitative reverse transcription PCR (qPCR) of total RNA were used with the Maxima SYBR Green qPCR (Thermo, Carlsbad, CA, USA) and the LightCycler (Roche, Basel, Switzerland). The sequences of the primers are as follows: forward: TTGCCCAAGCTATTCTCAGTCGA and reverse: CAGAGACACCGCCAGCCACAT for AQP9; and forward: GTGGGGCGCCCCAGGCACCA and reverse GTCCTTAATGTCACGCACGATTTC for β-actin. The ratio of each level to control represented the relative quantity of AQP9 mRNA expression. For Western blot analysis, briefly, total protein was separated on a precast 6% to 12% polyacrylamide gel and blotted with antibodies for AQP9, Bcl-2, Bax, Caspase 3, ERK1/2, p-ERK1/2 and GAPDH. Densitometric analysis of protein bands was performed via Image J software (NIH, Bethesda, MD, USA).

### 4.3. Small Interfering (Si) RNA Treatment

Small interfering RNA (siRNA) against AQP-9 was generated using the silencer siRNA (Santa Cruz sc-42371) according to the manufacturer’s recommendations. PC-3 cells were seeded (1.0 × 10^5^ cells/mL) onto a six-well plates, and incubated at 37 °C with 5% CO_2_ overnight. Cells were then transfected with siRNA using Lipofectamine 2000 (Invitrogen Life Technologies), according to the manufacturer’s instructions. The knockdown efficiency was tested 48 h later.

### 4.4. Cell Proliferation Assay, Wounding Healing Assay and Invasion Assay

For proliferation assay, different treatment cells were seeded onto 96-well plates (5000 per well) in F12 with 5% FBS and allowed to attach overnight. MTT was added at the following day. Cell proliferation assays were performed using a MTT cell proliferation and Cytotoxicty Detection Kit (Keygentec, Taoshan, China) according to the manufacturer’s instructions. Absorbance was measured at 550 nm using Mikrotek Laborsysteme (Mikrotek, Overath, Germany).

Invasion assays were performed with three groups. These cells were prepared in suspensions in culture medium containing 1 × 10^4^ cells for 24-well chambers. The BD BioCoat TM Matrigel Invasion Chambers (BD Biosciences, San Jose, CA, USA) were incubated for 24 h at 37 °C in a 5% CO_2_. After incubation, non-invading cells were removed from the upper side of the membrane by scrubbing.

After staining by 0.2% crystal violet, the invading cells were observed under the microscope at 200× magnification and counted in the four fields per experiments, and results were expressed in the form of a bar graph.

Wound healing assay was used as follows: a scratch was made using a micropipette tip and cells were washed with PBS (phosphate buffer saline) to remove detached cells and debris. Photographs of the same area of the wound were taken at 0, 24, and 36 h for measuring the closure of the wound after the treatment of the transfection.

### 4.5. Immunofluorescence Microscopy

The cells grown in chamber slides, then cells were fixed in 4% paraformaldehyde, and permeabilized using 0.2% Triton-X100 in PBS and blocked with 5% BSA/PBS, samples were stained with primary antibodies AQP9 (1:300, Santa Cruz Biotech) and then followed by rhodamine-conjugated secondary antibodies. Nuclei were counterstained with DAPI (4′,6-diamidino-2-phenylindole). Images were captured on a Leica TCS-SP8 fluorescence microscope (Leica, Wetzlar, Germany).

### 4.6. Statistical Analysis and Gene Set Enrichment Analysis (GSEA)

All experiments were repeated three times and data are expressed as the mean ± SD. Statistical analysis and graphs was performed with SPSS 17.0 (IBM, Armonk, NY, USA) and OriginPro 8.0 (Originlab, Northampton, MA, USA) using Student’s *t*-test (data on two groups) or analysis of variance and (LSD) *post hoc* test (data on three groups) analysis of variance *p* < 0.05 was considered to indicate a statistically significant difference. Prostate cancer dataset was downloaded from the NCBI Gene Expression Omnibus database, Access ID: GSE55945).

## 5. Conclusions

In conclusion, we found that AQP9 was expressed in prostate cancer and provided for the first time that AQP9 played a key role in the proliferation, apoptosis and metastasis of androgen-independent prostate cancer cells, and AQP9 might regulate these biological progress through ERK signal pathways and, thus, may provide useful information for further targeted therapy.

## Abbreviations

PCa	Prostate cancer
AQP	Aquaporin
siRNA	Small interfering RNA
ERK	Extracellular signal-regulated kinase
GSEA	Gene set enrichment analysis

## References

[1] Siegel R., Ward E., Brawley O., Jemal A. (2011). Cancer statistics, 2011: The impact of eliminating socioeconomic and racial disparities on premature cancer deaths. CA Cancer J. Clin..

[2] Sluka P., Davis I.D. (2013). Cell mates: Paracrine and stromal targets for prostate cancer therapy. Nat. Rev. Urol..

[3] Verkman A.S. (2005). More than just water channels: Unexpected cellular roles of aquaporins. J. Cell Sci..

[4] Bloch O., Papadopoulos M.C., Manley G.T., Verkman A.S. (2005). *Aquaporin-4* gene deletion in mice increases focal edema associated with staphylococcal brain abscess. J. Neurochem..

[5] Papadopoulos M.C., Verkman A.S. (2007). Aquaporin-4 and brain edema. Pediatr. Nephrol..

[6] Li Z., Li B., Zhang L., Chen L., Sun G., Zhang Q., Wang J., Zhi X., Wang L., Xu Z. (2016). The proliferation impairment induced by AQP3 deficiency is the result of glycerol uptake and metabolism inhibition in gastric cancer cells. Tumour Biol..

[7] Xu J.L., Xia R. (2014). The emerging role of aquaporin 5 (AQP5) expression in systemic malignancies. Tumour Biol..

[8] El Hindy N., Bankfalvi A., Herring A., Adamzik M., Lambertz N., Zhu Y., Siffert W., Sure U., Sandalcioglu I.E. (2013). Correlation of aquaporin-1 water channel protein expression with tumor angiogenesis in human astrocytoma. Anticancer Res..

[9] Wei M., Shi R., Zeng J., Wang N., Zhou J., Ma W. (2015). The over-expression of aquaporin-1 alters erythroid gene expression in human erythroleukemia K562 cells. Tumour Biol..

[10] Wang J., Tanji N., Sasaki T., Kikugawa T., Song X., Yokoyama M. (2008). Androgens upregulate aquaporin 9 expression in the prostate. Int. J. Urol..

[11] Nicchia G.P., Frigeri A., Nico B., Ribatti D., Svelto M. (2001). Tissue distribution and membrane localization of aquaporin-9 water channel: Evidence for sex-linked differences in liver. J. Histochem. Cytochem..

[12] Kawasaki H., Taira K., Morris K.V. (2005). siRNA induced transcriptional gene silencing in mammalian cells. Cell Cycle.

[13] Shankar E., Song K., Corum S.L., Bane K.L., Wang H., Kao H.Y., Danielpour D. (2016). Signaling Network Controlling Androgenic Repression of c-Fos in Prostate Adenocarcinoma Cells. J. Biol Chem..

[14] Wang X.Y., Hao J.W., Zhou R.J., Zhang X.S., Yan T.Z., Ding D.G., Shan L. (2013). Meta-analysis of gene expression data identifies causal genes for prostate cancer. Asian Pac. J. Cancer Prev..

[15] Papadopoulos M.C., Saadoun S. (2015). Key roles of aquaporins in tumor biology. Biochim. Biophys. Acta.

[16] Yang J.H., Yan C.X., Chen X.J., Zhu Y.S. (2011). Expression of aquaglyceroporins in epithelial ovarian tumours and their clinical significance. J. Int. Med. Res..

[17] Fossdal G., Vik-Mo E.O., Sandberg C., Varghese M., Kaarbø M., Telmo E., Langmoen I.A., Murrell W. (2012). AQP 9 and brain tumour stem cells. Sci. World J..

[18] Pastor-Soler N., Bagnis C., Sabolic I., Tyszkowski R., McKee M., van Hoek A., Breton S., Brown D. (2001). Aquaporin 9 expression along the male reproductive tract. Biol. Reprod..

[19] Pastor-Soler N., Isnard-Bagnis C., Herak-Kramberger C., Sabolic I., van Hoek A., Brown D., Breton S. (2002). Expression of aquaporin 9 in the adult rat epididymal epithelium is modulated by androgens. Biol. Reprod..

[20] Ge C., Zhao G., Li Y., Li H., Zhao X., Pannone G., Bufo P., Santoro A., Sanguedolce F., Tortorella S. (2016). Role of Runx2 phosphorylation in prostate cancer and association with metastatic disease. Oncogene.

[21] Liao A., Wang W., Sun D., Jiang Y., Tian S., Li J., Yang X., Shi R. (2015). Bone morphogenetic protein 2 mediates epithelial-mesenchymal transition via AKT and ERK signaling pathways in gastric cancer. Tumour Biol..

[22] Zhao P., Ma W., Hu Z., Zang L., Tian Z., Zhang K. (2016). Filamin A (FLNA) modulates chemosensitivity to docetaxel in triple-negative breast cancer through the MAPK/ERK pathway. Tumour Biol..

[23] Nico B., Ribatti D. (2010). Aquaporins in tumor growth and angiogenesis. Cancer Lett..

[24] Huo C., Kao Y.-H., Chuu C.-P. (2015). Androgen receptor inhibits epithelial-mesenchymal transition, migration, and invasion of PC-3 prostate cancer cells. Cancer Lett..

[25] Li W., Qi K., Wang Z., Gu M., Chen G., Guo F., Wang Z. (2015). Golgi phosphoprotein 3 regulates metastasis of prostate cancer via matrix metalloproteinase 9. Int. J. Clin. Exp. Pathol..

